# Mechanical properties and drug release behavior of PCL/zein coated 45S5 bioactive glass scaffolds for bone tissue engineering application

**DOI:** 10.1016/j.dib.2015.07.013

**Published:** 2015-07-23

**Authors:** Zeinab Fereshteh, Patcharakamon Nooeaid, Mohammadhossein Fathi, Akbar Bagri, Aldo R. Boccaccini

**Affiliations:** a Institute of Biomaterials, Department of Materials Science and Engineering, University of Erlangen-Nuremberg, Cauerstrasse 6, 91058 Erlangen, Germany; b Institute of Science, High Technology and Environmental Sciences, Graduate University of Advanced Technology, 76315117 Kerman, Iran; c Biomaterials Research Group, Department of Materials Engineering, Isfahan University of Technology, Isfahan 8415683111, Iran; d Dental Materials Research Center, Isfahan University of Medical Sciences, Isfahan, Iran; e Department of Materials Science and Engineering, Massachusetts Institute of Technology, Cambridge, MA 02139, USA

**Keywords:** Bioactive glass, Scaffolds, Mechanical properties, PCL/zein coating, Drug delivery

## Abstract

This article presents data related to the research article entitled “The effect of coating type on mechanical properties and controlled drug release of PCL/zein coated 45S5 bioactive glass scaffolds for bone tissue engineering” [1]. We provide data on mechanical properties, in vitro bioactivity and drug release of bioactive glass (BG) scaffolds coated by poly (*ε*-caprolactone) (PCL) and zein used as a controlled release device for tetracycline hydrochloride (TCH). By coating the BG scaffolds with PCL or PCL/zein blend the mechanical properties of the scaffolds were substantially improved, i.e., the compressive strength increased from 0.004±0.001 MPa (uncoated BG scaffolds) to 0.15±0.02 MPa (PCL/zein coated BG scaffolds). A dense bone-like apatite layer formed on the surface of PCL/zein coated scaffolds immersed for 14 days in simulated body fluid (SBF). The data describe control of drug release and in vitro degradation behavior of coating by engineering the concentration of zein. Thus, the developed scaffolds exhibit attractive properties for application in bone tissue engineering research.

Specifications table

**Table:** 

Subject area	Biology
More specific subject area	Drug delivery
Type of data	Figure
How data was acquired	FTIR spectrometer (Nicolet 6700), compressive strength (Zwick/RoellZ050 mechanical tester), contact angle measuring instrument (DSA30 Kruess), pH meter (pH 7.4, Sigma-Aldrich, USA), UV–vis spectrophotometer (Specord 40, Analytik Jena, Germany)
Data format	Analyzed
Experimental factors	BG scaffolds were fabricated by the foam replication method
Experimental features	PCL and zein solutions in different concentrations were prepared by dissolving PCL in chloroform and zein in ethanol. Then, scaffolds were coated by immersed in the solution.
Data source location	University of Erlangen-Nuremberg, Erlangen, Germany
Data accessibility	Data is supplied with this article.

Value of the data

• The data described here, PCL/zein composite as an organized innovative design coating, may be helpful to researchers who are interested in applications in in-situ drug release in bone tissue engineering.
• Composite PCL/zein coated BG scaffolds with a novel type of drug controlled-release are shown.
• Compressive strength and mechanical stability are shown to be significantly increased via the optimized method for dip coating the BG scaffolds by PCL and zein.

## 1 Data, experimental design, materials and methods

**Fig. 1:**
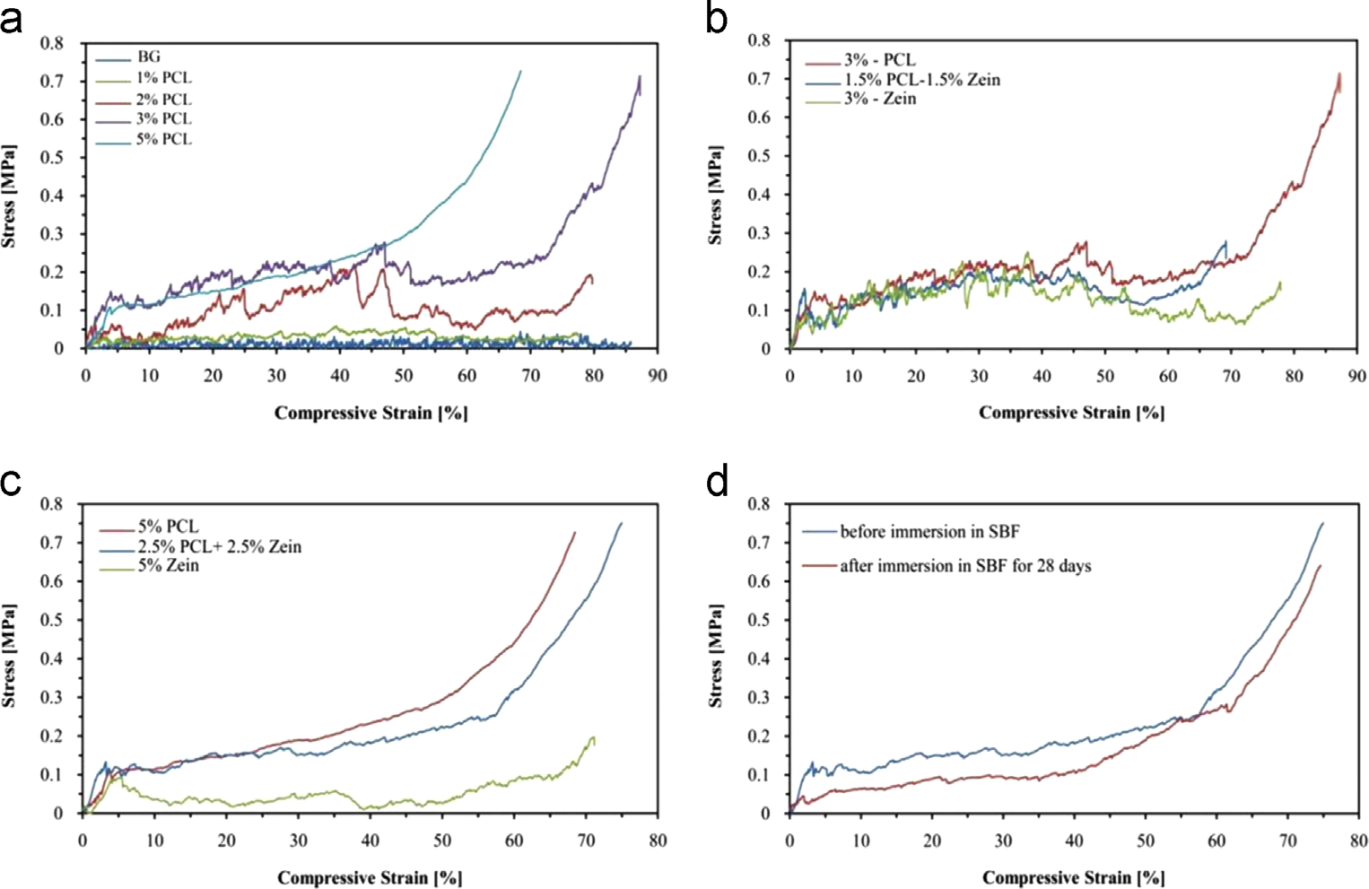
Effect of different concentration of (a) PCL, (b) PCL/zein composite coating with 3 wt% and (c) with 5 wt%, on the stress–strain behavior of BG-based scaffolds in compression. (d) Stress–strain curve of PCL/zein coated of BG-based scaffolds with 5 wt% of PCL/zein under compressive loads before and after immersion in SBF for 28 days.

**Fig. 2:**
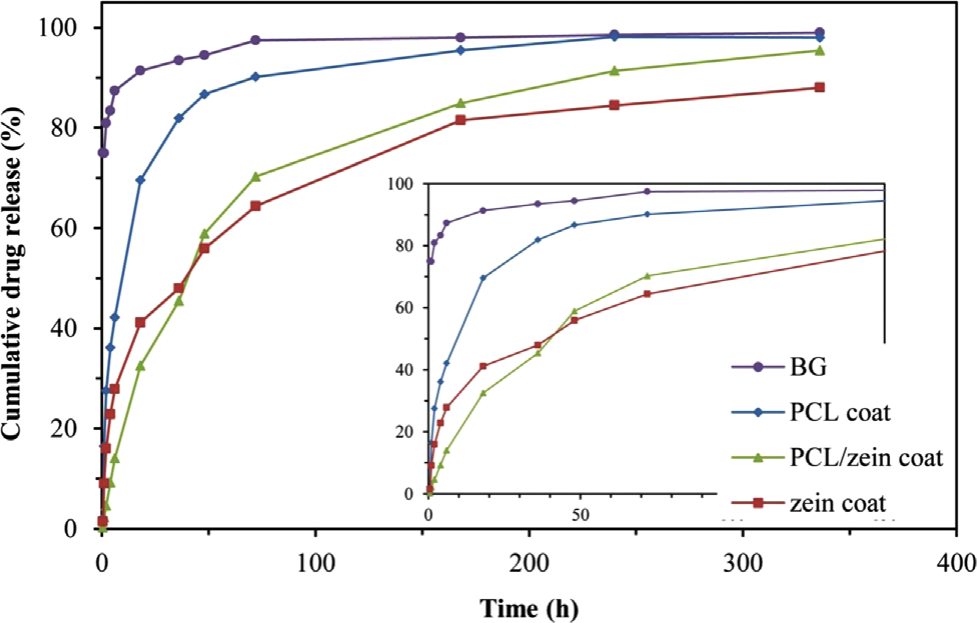
Released TCH from BG scaffolds of uncoated and coated with PCL, PCL/zein and zein TCH-loaded. The inserted curve indicates more detail of the release curve in the first 50 h.

The data provided here are stress–strain curves for uncoated and coated scaffolds with different concentration of PCL and zein before and after immersion in SBF for 28 days (Fig. 1) and drug release curves of BG scaffolds of uncoated and coated with PCL, PCL/zein and zein TCH-loaded (Fig. 2).

### 1.1 Fabrication of bioglass-based scaffolds

Fully reticulated polyurethane (PU) foams (45 ppi, Eurofoam, Germany) and 45S5 BG powder (composition in wt%: 45% SiO_2_, 24.5% Na_2_O, 24.4% CaO and 6% P_2_O_5_) of particle size ~5 µm have been used to fabricate the scaffolds. The scaffolds were fabricated by the foam replication method, following a similar process as described elsewhere [2].

### 1.2 Polymer coating procedure and drug loading

In order to obtain a dual PCL/zein coating, PCL/zein (Sigma-Aldrich, USA) (50:50% w/w) solution was prepared by dissolving PCL in chloroform and zein in ethanol (Merck, Germany). Then, scaffolds were coated by this solution for two times. For the drug loading together with the PCL/zein polymer, TCH (0.5% w/v) was dissolved in ethanol solution and the coating procedure was performed similarly as for the unloaded coatings [1].

### 1.3 Characterization of coated scaffolds

By coating the BG scaffolds with PCL or PCL/zein blend the mechanical properties of the scaffolds were substantially improved, i.e. the compressive strength increased from 0.004±0.001 MPa (uncoated BG scaffolds) to 0.15±0.02 MPa (PCL/zein coated BG scaffolds). The compressive strength values of the coated scaffolds are close to the lower bound strength value for spongy bone. The compressive strength of superficial of osteochondral tissue is 0.079 MPa [3], [4]. Fig. 1 shows typical compressive stress–strain curves for uncoated and coated scaffolds with different concentration of PCL and PCL/zein indicating the increase of both the compressive strength and the area under the stress displacement curve, which is related to the work of fracture (toughness of the material). By increasing the concentration of polymer, the compressive strength of coated scaffolds was increased. The SEM images of PCL, PCL/zein and zein-coated scaffolds showed a smooth surface of the struts and no large microcracks present on scaffolds coated by PCL. Based on this data, the scaffold coated using a solution of 2.5% PCL: 2.5% zein w/v (1:1) was chosen for further investigation. The porosity and the compressive strength of the present coated scaffolds were ~ 94% and 0.15 MPa, respectively.

### 1.4 Assessment of bioactivity of the PCL/zein coated scaffolds

Fig. 1d shows the mechanical behavior of the PCL/zein coated scaffolds before and after immersion in SBF for 28 days. The compressive strength of the scaffolds decreased markedly during 28 days of immersion in SBF (0.094±0.004 MPa). The presence of bone-like apatite on the uncoated and coated scaffolds was confirmed by XRD, SEM and FTIR analyses. It is suggested that the PCL/zein coating will positively affect the bioactive character of 45S5 BG scaffolds. Indeed a cell biology assessment of the present scaffolds, in comparison to similar polymer coated BG scaffolds reported recently [5], [6], [7] remains to be carried out. The weight loss data of scaffolds are also increased by increasing the amount of zein. The weight loss for the PCL/zein (50:50) and zein films were 16% and 39%, respectively. It suggests that a majority of the zein component degraded after 28 days of immersion.

### 1.5 Drug release analysis

The absorbed drug within the coated scaffolds was released in a sustained manner over 10 days (Fig. 2). Three trends were observed for the drug release profiles: rapid, very slow and controlled drug released. The coated BG scaffolds containing zein have reduced release level compared to the uncoated and the coated scaffolds without protein. This data suggest that the entrapment of the drug by zein protein played an important role in the release behavior. The formation of protein–drug interactions is likely to contribute to the kinetics of the process, which should be related to the establishment of hydrogen bonds, van der Waals forces, and the release of solvent molecules [8], [9]. The prediction of the actual mechanism is challenging. Some of the reasons that make such prediction difficult are related to the fact that the interactions also depend on the degradation rate of both components. In addition, there are difficulties for the evaluation of the enthalpy and entropic contribution of the solvent [9]. ATR-FTIR spectra of the TCH-loaded scaffolds are shown in the Supplementary data. To shed some light on the release rate of TCH from PCL/zein coated scaffolds a logarithmic function is fit to the experimental data. The function that we fit is *D=*17.9 ln(*t*)−4.4785; where *D* is the cumulative drug release percentage and “*t*” is the release time in hours. The analysis of the data demonstrated that the total amount of TCH in PCL/zein coated scaffolds can be released after less than 15 days, as observed in previous researches [5], [7], [10].

## Footnotes

Appendix A Supplementary data associated with this article can be found in the online version at doi:10.1016/j.dib.2015.07.013.
