# Supplementary figures and images for: Mechanical properties and drug release behavior of PCL/zein coated 45S5 bioactive glass scaffolds for bone tissue engineering application
Source: Data Brief. 2015 Jul 23;4:524–8. doi: 10.1016/j.dib.2015.07.013 (PMC4783522; doi:10.1016/j.dib.2015.07.013)

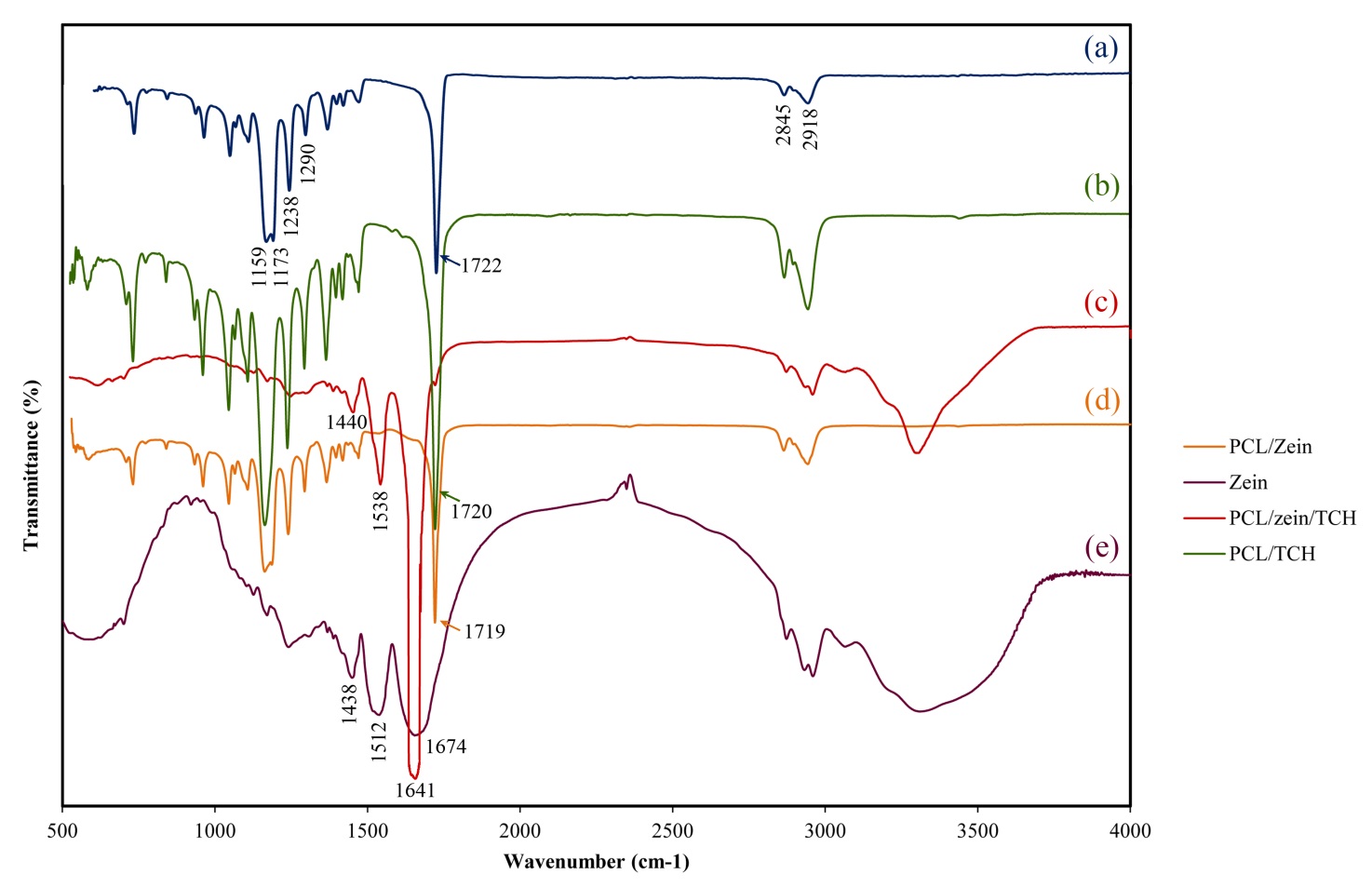


FTIR spectra of: (a) PCL, (b) TCH - loaded PCL, (c) TCH - loaded PCL-zein, (d) PCL-zein and (e) zein.

Supplement: Supplementary file 1 — Supplementary data [file mmc1.docx]
